# Cell Surface Concentrations and Concentration Ranges for Testing *In Vitro* Autocrine Loops and Small Molecules

**DOI:** 10.1371/journal.pone.0051796

**Published:** 2012-12-28

**Authors:** Nikhil Mittal

**Affiliations:** Cardiovascular Research Center, Massachusetts General Hospital, Boston, Massachusetts, United States of America; Semmelweis University, Hungary

## Abstract

A common assumption made when performing *in vitro* cellular assays is that the concentration of substances in the culture system is uniform. However, since the cells that internalize and secrete substances reside at the bottom of the well, it is conceivable that a concentration gradient could arise across the fluid layer. Importantly, the concentration of a substance in the vicinity of a cell, which is the concentration of interest, cannot be measured via existing methods. In this work a simple strategy for estimating the concentration of a chemical species at the surface of a cell is presented. Finally, this result is used to outline a method for determining the appropriate concentration ranges for testing *in vitro* autocrine loops and small molecules.

## Introduction

**Figure 1:**
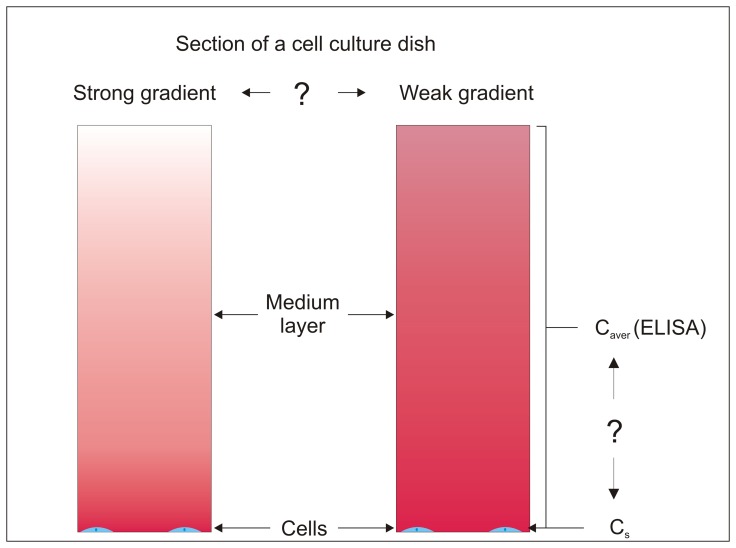
Schematic of the problem under consideration. The relation between the ligand concentration at the cell surface (C_s_) and the average concentration in the medium (C_aver_ which can be measured using ELISA) is related to whether there is a strong or weak gradient across the medium layer above the cells that are the source of the ligand.

A common assumption made when performing *in vitro* assays is that the concentration of substances in the culture system is uniform. However, since the cells that internalize and secrete molecules reside at the bottom of the well, it is conceivable that a concentration gradient could arise across the fluid layer (Figure 1). While the culture medium can be assayed to determine the concentration of any of its constituents, such measurements only provide the average concentration. The concentration of a substance in the vicinity of a cell, which is the concentration of interest, cannot be measured via existing techniques. The concentration of a chemical species at the surface of a cell is important because it is this concentration that determines the binding and internalization rates; binding/internalization rate  =  rate constant x surface concentration. One solution to this problem, which is the one used in this manuscript, is to use mathematical tools to find a relation between the cell surface concentration and the average medium concentration. Previous work on the *in vitro* transport of autocrine ligands has focused on determining the fraction of secreted ligand that is captured by the same cell itself (i.e. the one that secreted the captured molecule) [1], [2], and the average concentration of the ligand in the culture [3], [4]. However, they did not examine concentration gradients and the cell surface concentration.

In this study, two cases are examined: 1) in which a substance, typically a protein, is taken up *and* produced by cells i.e. autocrine signaling. When testing for *in vitro* autocrine loops, the amount of ligand added to the culture is usually arbitrary and, as will be demonstrated, therefore often non-physiological. This study provides a method for determining a physiological concentration range. 2) The second case examined is the case in which a substance (such as a small molecule) is only taken up by cells.

## Results

**Figure 2:**
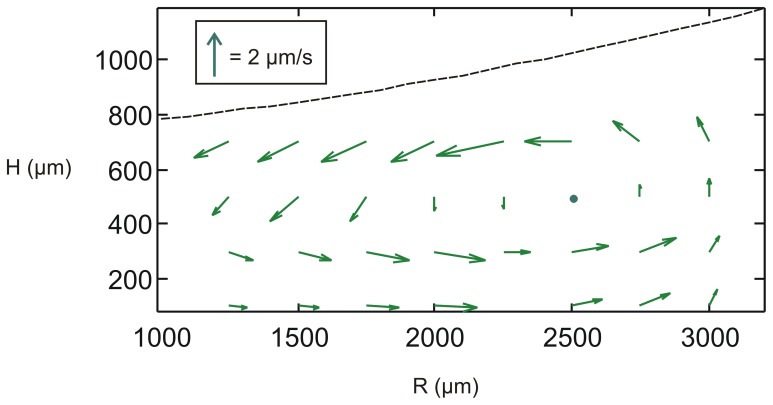
Steady-state flow pattern in a well within a 96-well plate.

In general, the relationship between the average concentration and concentration near the cell surface will depend on the type of mass transport in the culture dish. Measurements of flow in a single well of a 96-well plate led to the observation that after an initial transient that lasts for about 2 hours, a single vortex emerges, with a maximum velocity of O(1 µm/s) (Figure 2). This system is challenging to solve exactly, but I demonstrate that even in the presence of diffusion alone, the concentration gradient is typically shallow.

### Autocrine signaling

Let us first study the case in which a chemical species is both – produced and internalized by cells, namely, autocrine signaling. Autocrine signaling plays a role in physiological processes such as cell growth [5] and differentiation [6]. *In vitro* demonstration of the existence of an autocrine “loop” requires proof that the factor is produced by the cell, and that the cell shows altered physiology in response to changes in the total amount of bound ligand. The former is usually accomplished by analyzing medium conditioned by cells, while the latter is achieved either via receptor knockdown/knockout/inhibition, or by adding ligand to cell cultures. Receptor knockdown/knockout/inhibition is often challenging because (i) there may be several receptors for a ligand (ii) the identity of some or all receptors may be unknown and (iii) a single receptor may bind several ligands. Thus, a large percentage of studies (also) use the second option, which is to demonstrate a cellular response upon addition of exogenous ligand to cultures. However, *the amount of ligand added is usually arbitrary* and such studies may suffer from the lack of quantitative agreement between the amount of ligand available to a cell in culture, and the amount required to produce a physiological response. As a concrete example, if the maximum concentration of an autocrine ligand in the vicinity of a cell is 10 ng/ml under normal culture conditions, but it is found that 100 ng/ml of exogenously added ligand is required to produce a response, then this autocrine signal is not modulating the response under consideration (under the above culture conditions).

In this section a relation between the concentration at the cell surface and the average concentration in the culture is obtained. This relation, along with a measurement of the average concentration of the factor in a culture (using, say, an enzyme-linked immunosorbent assay (ELISA) or quantitative mass spectrometry), can be used to determine the cell surface concentration of an autocrine ligand, and thus, the appropriate range of concentrations that needs to be tested in the above type of assay. While previous studies have examined the *in vitro* transport of autocrine ligands [1], [2], [3], [4], they did not examine the cell surface concentration. Additionally, they did not consider the case in which a substance is added to the culture, which is discussed below.

**Figure 3:**
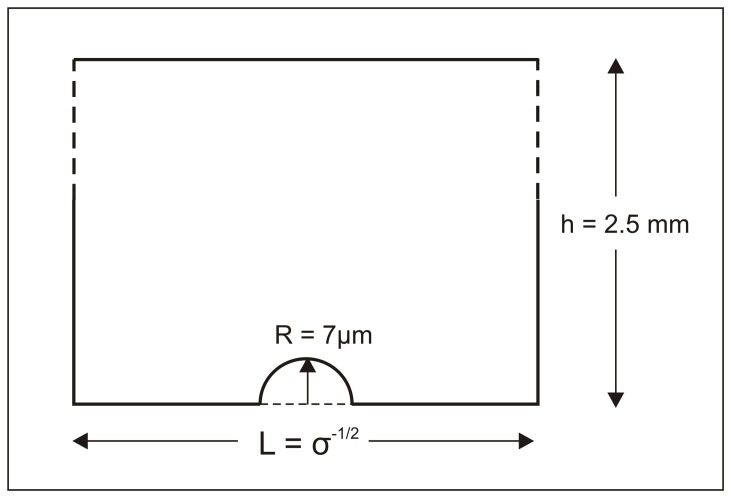
Geometry used in the simulation. The cell was modeled as a hemisphere with a radius of 7 μm. The height of the medium layer was 2.5 mm. The cell density was varied by varying the value of the length of the square base.

Numerical modeling was used to calculate the ratio of the cell surface concentration to the average medium concentration (α). The geometry used in the simulations is shown in Figure 3. I.e. cells were modeled as having a radius of 7 μm; the medium layer was taken to be 2.5 mm in height, which is fairly typical; and the distance between adjacent cells (L) depends on the plating density.

**Figure 4:**
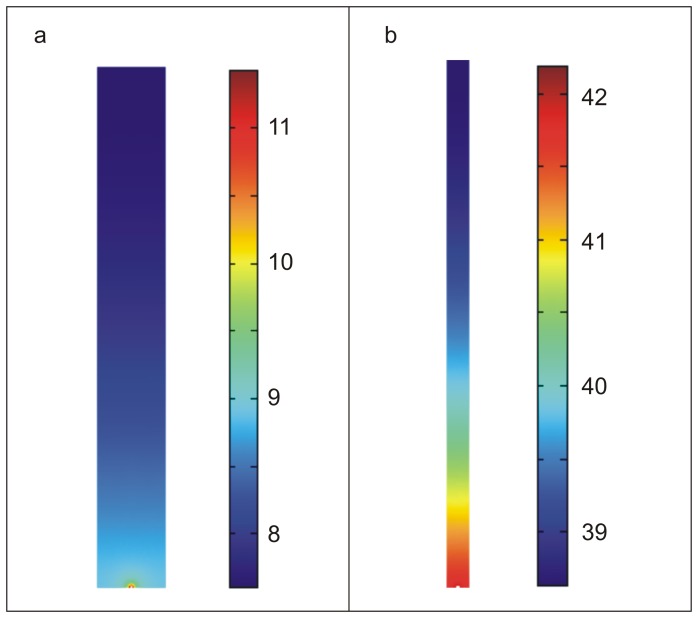
Concentration profiles obtained from the simulation for 1000 **cells/cm^2^** (**a**) **and 10,000 cells/cm^2^** (**b**) **at t = 48** **hours.** Concentrations are in units of pM. For this data, *r* = 5×10^−13^ moles/m^2^/s (approximately 10 molecules/cell/s), *k_on_* = 10^8^ 1/(M.min), *R* = 10^5^ receptors per cell, diffusion co-efficient  = 10^−10^ m^2^/s. More detailed models are described in the supporting information (Text S2, Text S3).

**Figure 5:**
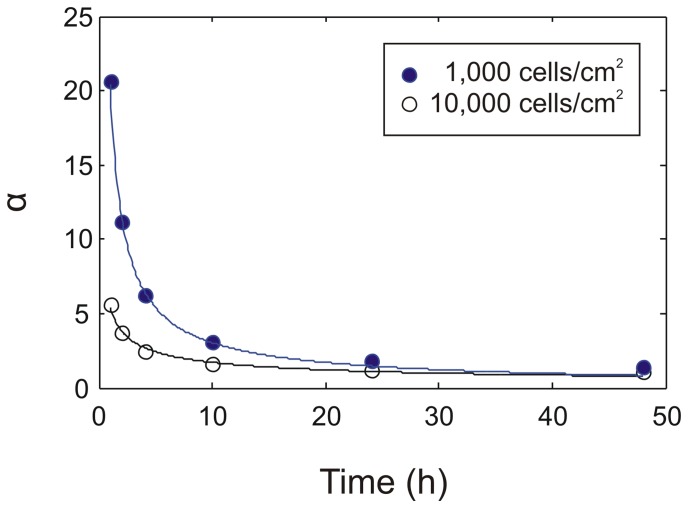
The concentration gradient of the ligand as a function of time for two cell densities. Circles are obtained from the simulation; the lines are guides to the eye. For this data, the secretion rate was 5×10^−12^ moles/m^2^/s, *k_on_* = 10^8^ 1/(M.min), 10^5^ receptors per cell, and the diffusion co-efficient was 10^−10^ m^2^/s. More detailed models are described in the supporting information (Text S2, Text S3).

For the parameter values for this process, we initially started with values for the EGF system [2], [7], and varied all parameters by a factor of 100 (Supporting Information: Text S1). Cell densities of 10^3^–10^4^ cells/cm^2^ were used (results are applicable to adherent and non-adherent cells). As shown in Text S1, the concentration gradient is inversely related to the cell density. Thus the values of the gradients shown here are higher than those in typical cultures, and provide an upper bound. Typical concentration profiles obtained from the simulations are shown in Figure 4. For typical culture densities (>1000 cells/cm^2^), the ratio of the concentration at the cell surface to the average concentration (α) always decays rapidly, and is typically less than 3 by 24 hours after the beginning of the culture (Figure 5, Text S1, Text S2, Text S3) for a wide variety of conditions (see below). I.e., the concentration of the ligand at the surface of a cell lies between the average value in the medium, and three times this value, with the additional requirement that the medium is conditioned for at least 24 hours before making the average concentration measurement. This result is not surprising given that the diffusion time for a typical cytokine, which are small proteins, for a distance of 1 mm, which is typically half the height of the fluid layer in a cell culture, is only about 3 hours. Indeed, it can be shown that this result is independent of the specifics of the binding stoichiometry and kinetics (Text S3). Thus, for efficacy testing of autocrine signaling, it is sufficient to test the concentration range between the average concentration (at t>24 h), and 3 times this concentration, which covers the range of possible cell surface concentrations of the ligand.

Finally, let us examine how the use of this criterion will significantly reduce the number of false-positive interactions obtained via use of this assay with the aid of an example. In a recent study [8] the authors used an ELISA to demonstrate that the concentration of a factor in the culture was less than 200 pg/ml (t>24 h). However, when they tested the effect of the factor on cell phenotype, they added the factor at a concentration of 100 ng/ml, i.e. 500-fold higher. However, the analysis presented in this work demonstrates that if the average concentration is 200 pg/ml, the cell surface concentration cannot exceed 600 pg/ml. Thus, any effects seen at higher concentrations are non-physiological with regard to autocrine signaling. Thus the use of this criterion will significantly reduce the number of false-positive interactions obtained via use of this assay.

### Substance added to a culture

Next let us consider the case in which a substance added to a culture is internalized by cells. A commonly performed biological assay is to measure an appropriate cellular response upon addition of an exogenous substance to a culture. However, as a result of cellular uptake, the concentration near the cells could differ from the supplied concentration (Figure 1). The value of the gradient is determined by competition between cellular uptake, which increases the gradient, and diffusion, which decreases the gradient. As in the previous case, numerical modeling was used to calculate the ratio of the cell surface concentration to the average medium concentration (α).

Let us examine the values of α obtained from the model for a low value of cell density (1000 cells/cm^2^), which results in the largest gradients (Text S1). For chemical species with a diffusion constant of 10^−9^ m^2^/s at 37°C (typically small molecules with a molecular weight of around 200 Da), the gradient is indeed small, with 0.9<α_24_<1 (where α_24_ is the ratio of the cell surface concentration to the average medium concentration at 24 hours after feeding/passaging) as long as at least ∼50% of the small molecule is still retained in the medium (Table 1). I.e. the cell surface concentration can differ from the average concentration by only 10% at the most for low to moderate uptake rates.

**Table 1:** Values of α_24_ for a plating density of 1000 cells/cm^2^ and diffusion coefficient 10^−9^ cm^2^/s.

*k* (s^−1^)	α_24_	c_24_/c_0_
1e-6	0.99	0.91
1e-5	0.92	0.44
1e-4	0.53	0.025

The uptake rate was taken to be *kc_s_*, where *c_s_* is the concentration of the added substance at the surface of the cell. c_0_ is the initial (uniform) concentration, and c_24_ is the final average concentration.

### Validity and Applicability of the Model

The validity and applicability of the model is worthy of discussion. (i) With regard to autocrine signaling, this work only pertains to the concentration range that needs to be tested in order to determine whether autocrine signaling is active in *in vitro* cultures. The maximum physiological concentration at a cell's surface *in vivo* will depend on a cell's microenvironment and therefore a result that is broadly applicable cannot be derived. However, an improved understanding of *in vitro* autocrine signaling could have important applications in cell expansion and differentiation for cell and cell-derived therapies, and consequently, in tissue engineering (Table S1). (ii) In the model used, diffusion through the layer of extracellular matrix (ECM) that typically surrounds cells was not considered. However, studies have shown that the co-efficient of diffusion of typical cytokines through the ECM is 10^−7^ cm^2^/s [9], [10], and that the thickness of ECM layers in cultures is typically less than 1 µm [11]. Thus the diffusion time through the ECM layer is less than 30 seconds, which is much less than the diffusion time through the liquid (∼3 hours) described above. Therefore diffusion through the ECM will not significantly alter the gradient of secreted factors. However, *binding* to the ECM could result in a difference between the ligand concentration at the cell surface, and that in the medium. Therefore these results may not apply to ECM-binding factors such as FGF-1 and FGF-2 [12]. (iii) Protein decay was not included in the model, which is equivalent to assuming that the half-life of the ligand in the medium is much greater than the diffusion time of 3 hours (say 24 hours or more). While half-life data for cytokines in culture medium are scarce [13], this appears to be true for a wide variety of cytokines in serum [14], [15], [16], [17]; most of them appear to have half-lives of several days to weeks. (iv) To demonstrate that autocrine signaling does *not* modulate the phenotype under consideration, in addition to showing a lack of response in the above concentration range, it is necessary to show a presence of response at some (higher) concentration. Assuming that ligand and receptors are expressed, a complete lack of response at all ligand concentrations suggests saturation of the pathway response. Trivially, this could be the result of performing the ligand addition assay at too high a cell density, such that the amount of ligand secreted by the cells themselves rapidly reaches the saturation value. However, if the complete lack of response persists at lower cell densities, then this would suggest that autocrine signaling is present, and that each cell in the culture captures that amount of self-secreted ligand (i.e. secreted by that very same cell itself) that results in saturation of the pathway response (Figure S1). (v) Finally, as described in the previous section, when testing the concentration range obtained using this method, it is possible that the supplied concentration could differ from the cell surface concentration. However, as described there, this difference correlates with depletion of the supplied ligand. Thus, it is necessary and sufficient to measure the average value of the ligand at the end of the assay to ensure that the ligand concentration has not changed by more than ∼10%. This would imply that the cell surface concentration did not differ from the supplied concentration by more than ∼10% (Text S3).

## Conclusions

The concentration of a substance in the vicinity of a cell, which is often a concentration of interest to biochemists, cannot be measured via existing methods. One solution to this problem, which is the one used in this manuscript, is to use mathematical tools to find a relation between the cell surface concentration and the average medium concentration. While previous studies have examined the *in vitro* transport of autocrine ligands [1], [2], and the bulk ligand concentration [3], [4], they did not examine such gradients. Additionally, they did not consider the case in which a substance is added to the culture, which is discussed in this work.

In this work it was demonstrated that for typical culture densities (>1000 cells/cm^2^), the ratio of the concentration at the cell surface to the average concentration (α) always decays rapidly, and is typically less than 3 by 24 hours after the beginning of the culture (Figures 5, Text S1, S2, S3). This is not surprising given that the diffusion time for a typical cytokine, which are small proteins, for a distance of 1 mm, which is typically half the height of the fluid layer in a cell culture, is only about 3 hours. The average concentration can be measured using ELISA, and thus an estimate of the cell surface concentration can be obtained. As a corollary, when testing putative autocrine factors, they should be tested at concentrations similar to that at the cell surface, i.e., less than 3 times the average concentration obtained by ELISA. Exceptions to this estimate exist, and are described in the results section.

Similarly, for small molecules added to a culture, the cell surface concentration can differ from the average concentration by only 10% at the most for low to moderate uptake rates. For larger molecules a more detailed analysis that includes convective transfer is required.

## Methods

### Numerical modeling

Numerical modeling of autocrine/paracrine signaling was performed using the Diffusion (time-dependent) module in COMSOL Multiphysics. The geometry used is shown in Figure 3. The effect of plating density (σ) is included in the model by varying the parameter L (Figure 3). The height of the liquid layer was 2.5 mm, which is fairly typical for cell culture. The cell was modeled as a hemisphere with a radius of 7 μm, which is typical for mouse embryonic stem cells (unpublished data), red blood cells, and typical fibroblasts [18].

The geometry was meshed using the default mesh parameters to generate a triangular mesh with 1696 points. For one set of parameter values I checked that increasing the number of mesh points by a factor of 10 led to a 0.07% change in the highest factor concentration and a 0.08% change in the lowest factor concentration. Thus the original number of mesh points (1696) is adequate for computing factor concentrations. The default solver was used, which is GMRES with an algebraic preconditioner, and default timesteps, with a maximum timestep of one hour.

It was assumed that the transport of substances is purely diffusive, i.e. that there is no convection in the dish. The presence of convection will reduce the concentration gradients. Thus, by making this assumption an upper bound on the concentration gradient is obtained.

The diffusion constant of autocrine factors in the medium was taken to be 0.5–1×10^−10^ m^2^/s which is typical for proteins in the 5–40 kDa range [19]; most diffusible proteins are in this mass range. No-flux boundary conditions were applied on all surfaces, except the hemisphere corresponding to the cell. For most of the simulations (see below), for this surface, the normal flux of the protein was set to *r – k_on_Rc_s_*, where *r* is the secretion rate, *k_on_* is the binding constant, *R* is the number of receptors per cell, and *c_s_* is the concentration of factor at the cell surface (*c_s_* is solved for within the simulation). It was assumed that the number of free receptors is constant. Previous work by Lauffenburger and colleagues [2] has shown that the receptor number reaches a steady state within 2 hours after the commencement of culture. Therefore this assumption is justified. A model that includes unbinding events and receptor dynamics was also briefly investigated (Text S2). The values for the above parameters were taken from Shvartsman *et* *al*. [2] (see Figure 4 in that paper): secretion rate (*r*): 1–1000 molecules/cell/second; binding constant (*k_on_*): 10^8^ M^−1^ min^−1^ (tested 10^7^–10^9^ M^−1^ min^−1^); number of receptors per cell (*R*): 10^5^ (tested 10^4^–10^6^). Finally, the model has one geometric parameter – the area of the bottom surface, which is the inverse of the plating density.

### Flow measurements

The flow pattern was reproducible across three experiments. 2 μm diameter fluorescent polystyrene beads were added to an (approximately) 7% NaCl solution to make them neutrally buoyant. 30 μl of this solution was pipetted into a well of a 96-well plate (Nunc, Rochester, NY). The plate was placed on a microscope stage (Axiovert 200, Carl Zeiss MicroImaging, Inc., Thornwood, NY) at room temperature. To minimize evaporation, 2–3 rows and columns of wells around the well of interest were filled with phosphate buffered saline (Invitrogen). Imaging was performed using a Spot RT Color camera (Diagnostic Instruments, Inc., Sterling Heights, MI). The beads were bright enough to be visualized without the need of a UV or halogen source i.e. they could be imaged using ambient lighting. This prevented additional flows in the well due to uneven heating of the liquid.

Stage height was controlled using Metamorph software (Molecular Devices). Bead centers were also tracked using Metamorph. Vertical velocities are approximate, and were determined using a manual procedure. Briefly, first the direction of the flow (up versus down) at a location was determined by tracking individual beads manually. Following this, a bead was imaged until it came into focus; the stage was moved up or down depending on the expected bead trajectory and bead imaging was continued until the bead again came into focus. The image in which the size of the bead was minimum was chosen to be the image corresponding to best focus. Bead image sizes were also measured using Metamorph. The distance moved by the stage was divided by the time for the bead to reappear in focus to obtain the vertical velocity.

## Supporting Information

Figure S1 **Schematic of the protocol for testing for autocrine signaling and interpreting the results obtained.**
(TIF)

Table S1 **Examples of studies that have implicated autocrine signaling in the expansion and differentiation of various types of cells that could be used for cell-based therapies.**
(PDF)

Text S1 **Effects of parameter variation on the concentration gradient.**
(PDF)

Text S2 **Model with receptor dynamics.**
(PDF)

Text S3 **Independence of results from binding stoichiometry and kinetics.**
(PDF)

Text S4 **Slowly diffusing substance added to a culture.**
(PDF)
