# Supplementary figures and images for: Cell Surface Concentrations and Concentration Ranges for Testing In Vitro Autocrine Loops and Small Molecules
Source: PLoS One. 2012 Dec 28;7(12):e51796. doi: 10.1371/journal.pone.0051796 (PMC3532204; doi:10.1371/journal.pone.0051796)

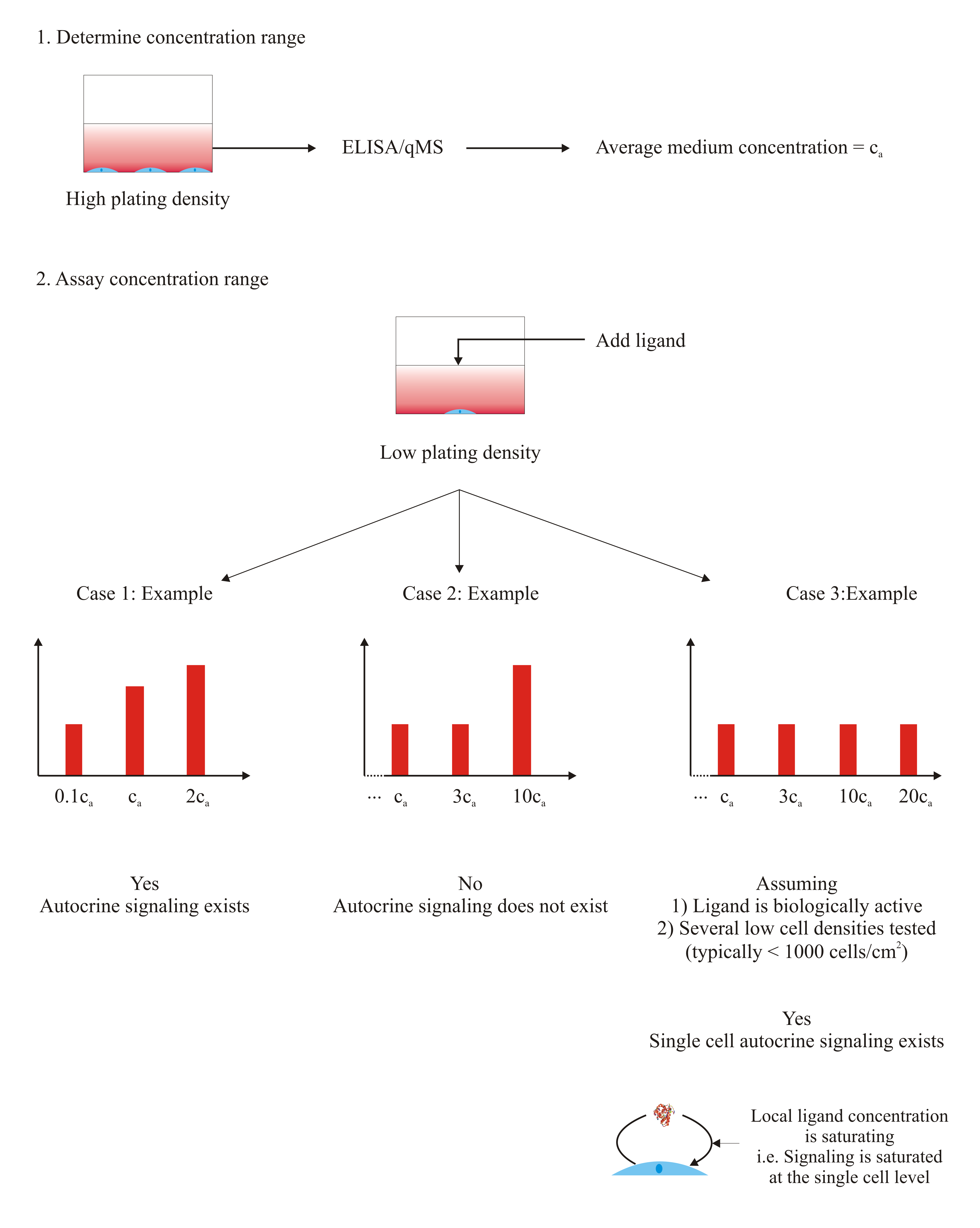

Supplement: Figure S1 — Schematic of the protocol for testing for autocrine signaling and interpreting the results obtained. (TIF) [file pone.0051796.s001.tif]
