# Supplementary material for: Cell Surface Concentrations and Concentration Ranges for Testing In Vitro Autocrine Loops and Small Molecules
Source: PLoS One. 2012 Dec 28;7(12):e51796. doi: 10.1371/journal.pone.0051796 (PMC3532204; doi:10.1371/journal.pone.0051796)
Supplement: Table S1 — Examples of studies that have implicated autocrine signaling in the expansion and differentiation of various types of cells that could be used for cell-based therapies. (PDF) [file pone.0051796.s002.pdf]

Table S5. Below are some examples of studies that have implicated autocrine signaling in the expansion and differentiation of various types of cells that could be used for cell-based therapies.

Expansion

Autocrine and Paracrine Angiopoietin 1/Tie-2 Signaling Promotes **Muscle Satellite Cell** Self-Renewal. Cell Stem Cell 5:298-309 (2009)

Cell Density Plays a Critical Role in Ex Vivo Expansion of **T Cells** for Adoptive Immunotherapy. Journal of Biomedicine and Biotechnology Article Number:386545 (2010)

Nodal Signaling via an Autocrine Pathway Promotes Proliferation of Mouse **Spermatogonial Stem/Progenitor Cells** Through Smad2/3 and Oct-4 Activation. Stem Cells 27:2580-2590 (2009)

Nodal Signaling Regulates the Bone Morphogenic Protein Pluripotency Pathway in Mouse **Embryonic Stem Cells.** Journal of Biological Chemistry 285:19747-19756 (2010)

Autocrine/paracrine platelet-derived growth factor regulates proliferation of **neural progenitor cells.** Cancer Research 66:8042-8048 (2006)

Autocrine fibroblast growth factor 2 signaling is critical for self-renewal of **human multipotent adipose-derived stem cells.** Stem Cells 24:2412-2419 (2006)

Influence of PI-3K/Akt pathway on Wnt signalling in regulating **myeloid progenitor cell** proliferation. Evidence for a role of autocrine/paracrine Wnt regulation. British Journal of Haematology 146:637-651 (2009)

Differentiation

Interplay between FGF2 and BMP controls the self-renewal, dormancy and **differentiation of rat neural stem cells**. Journal of Cell Science 124:1867-1877 (2011)

Hedgehog serves as a mitogen and survival factor during **embryonic stem cell neurogenesis.** Stem Cells 26:1097-1108 (2008)

Autocrine Regulation of Interferon gamma in Mesenchymal Stem Cells Plays a Role in Early **Osteoblastogenesis.** Stem Cells 27:550-558 (2009)

Autocrine production of TGF-beta 1 promotes **myofibroblastic differentiation of neonatal lung mesenchymal stem cells.** American Journal of Physiology – Lung Cellular and Molecular Physiology 298:L735-L743 (2010)
