# Supplementary material for: Cell Surface Concentrations and Concentration Ranges for Testing In Vitro Autocrine Loops and Small Molecules
Source: PLoS One. 2012 Dec 28;7(12):e51796. doi: 10.1371/journal.pone.0051796 (PMC3532204; doi:10.1371/journal.pone.0051796)
Supplement: Text S1 — Effects of parameter variation on the concentration gradient. (PDF) [file pone.0051796.s003.pdf]

Text S1: Effects of parameter variation on the concentration gradient

In Table S1 the values of the ratio of the cell surface ligand concentration to the average ligand concentration in the culture at t = 48 hours (α_{48}) for two plating densities as a function of k_{on} are shown. Results at 48 hours are reported because medium used for measuring ligand concentrations using ELISA is typically conditioned for at least 48 hours. As shown in Figure 5, the value of α for t > 48 hours will be smaller. One would expect α to be inversely correlated with density; for example, the concentration from a point source drops off as a^{-3}, where a is the distance from the source; but if there are "several" point sources arranged to form a line source, then the concentration only drops off as -log(a). α was calculated for two plating densities: 1000 cells/cm^2 and 10,000 cells/cm^2 to test that α is indeed higher at the lower plating density (Table S1). Maximal activation of an autocrine loop will occur in a confluent culture, which will have the highest factor concentrations. So the values corresponding to a plating density of 10,000 cells/cm^2 can be taken to be an upper bound on the values of α corresponding to maximal activation.

Table S1: Values of α_{48} for two plating densities and a range of k_{on} values. $R = 10^5$ receptors/cell, secretion rate = $5x10^{-13}$ moles/m^2/s, diffusion co-efficient = 10^{-10} m^2/s. α_{48} is the ratio of the cell surface ligand concentration to the average ligand concentration in the culture at t = 48 hours.

k_{on} ($M^{-1}min^{-1}$)	Plating density (cm^{-2})	
	1000	10000
10^7	1.46	1.13
10^8	1.42	1.06
10^9	1.26	1.01

Not surprisingly, while changes in the secretion rate (r) lead to a change in the actual concentration, the ratio α is independent of r (not shown). Next, let us look at the effects of varying k_{on} and the receptor number (R) (Table S2). Both these parameters affect the flux in a similar manner, since the net flux is $r - k_{on}Rc$ (see methods). Note that α_{48} is less than 2 for all values.

Table S2: Values of α_{48} for a range of k_{on} and R values, for 10000 cells/cm^2, secretion rate = $5x10^{-13}$ moles/m^2/s, and diffusion co-efficient = 10^{-10} m^2/s.

k_{on} ($M^{-1}min^{-1}$)	R (No. of receptors per cell)		
	10^4	10^5	10^6
10^7	1.15	1.13	1.06
10^8	1.13	1.06	1.01
10^9	1.06	1.01	1.003

Finally, the dependence of α on the diffusion co-efficient is shown in Table S3 (Text S3).
