# Supplementary material for: Cell Surface Concentrations and Concentration Ranges for Testing In Vitro Autocrine Loops and Small Molecules
Source: PLoS One. 2012 Dec 28;7(12):e51796. doi: 10.1371/journal.pone.0051796 (PMC3532204; doi:10.1371/journal.pone.0051796)
Supplement: Text S2 — Model with receptor dynamics. (PDF) [file pone.0051796.s004.pdf]

Text S2: Model with receptor dynamics

In Figure 5 the time-dependence of the ratio (α) of the secreted protein concentration at the surface of the cell to the average concentration in the culture was shown. Those values were obtained from a model where the number of receptors per cell was fixed at 10^5/cell and the off-rate was neglected. A model with receptor dynamics was also briefly investigated. For the model, values from Shvarstman *et al.* (Biophysical Journal 81, 1854-1867 (2001)), which correspond to the epidermal growth factor system, were used:

Surface complex dissociation rate (commonly called k_{off}) = $2x10^{-3}$ s^{-1}
Endocytosis rate for bound receptors = 10^{-3} s^{-1}
Internalization rate for unbound receptors = 10^{-4} s^{-1}
Receptor production rate = 10^{-12} moles/m^2/s

To model the receptor dynamics, in addition to the Diffusion module in COMSOL Multiphysics, two weak form (boundary) modules were used to simulate the dynamics of bound and unbound receptors. In the simulation the unbound receptor number was actually found to vary over the duration of the simulated culture (48 hours).

Figure S2 shows that the presence of receptor dynamics provides only a small correction to the values of α. For these simulations k_{on}=10^8 $M^{-1}min^{-1}$, r = $5x10^{-13}$ moles/ m^2/s, a cell density of 10^4 cells/cm^2, diffusion co-efficient = 10^{-10} m^2/s, and initial receptor density R_0=10^{-9} moles/m^2 ($1.85x10^5$ receptors/cell) were used.

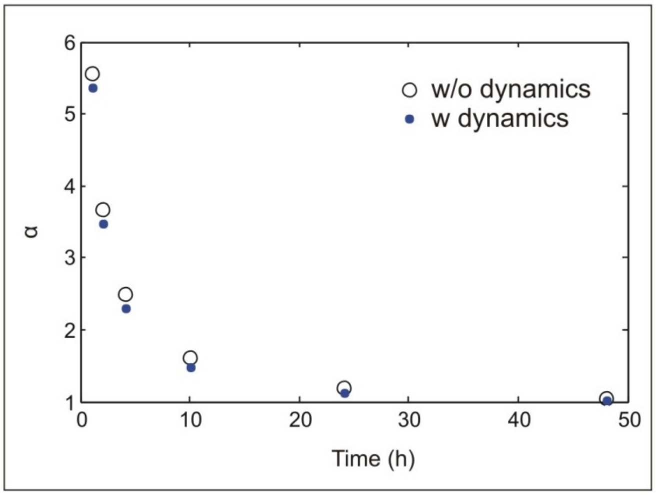

Figure S2: Alpha versus time from models with and without receptor dynamics.
