# Supplementary material for: Cell Surface Concentrations and Concentration Ranges for Testing In Vitro Autocrine Loops and Small Molecules
Source: PLoS One. 2012 Dec 28;7(12):e51796. doi: 10.1371/journal.pone.0051796 (PMC3532204; doi:10.1371/journal.pone.0051796)
Supplement: Text S3 — Independence of results from binding stoichiometry and kinetics. (PDF) [file pone.0051796.s005.pdf]

Text S3: Independence of results from binding stoichiometry and kinetics

We begin by hypothesizing that the concentration gradient is largest in the absence of binding. To gain an intuitive understanding of why this may be so, let us first observe that the concentration will always be maximum near the cell, which is the ligand source. In the presence of binding the cell will "consume" some of the ligand at its surface, decreasing this maximum value, and thereby reducing the gradient.

This hypothesis was tested using simulations and found to be true. For e.g., for a plating density of 10,000 cells/cm^2 and k_{on} = 10^8 $M^{-1}min^{-1}$, at t = 48 hours the value of α (cell surface concentration/average concentration) is 7% greater for the non-binding case as compared to the binding case. Observe also that in Table S2, the values of α_{48} increase with decrease in the receptor number and/or k_{on}.

Thus, an upper bound on α can be obtained by studying the non-binding case. As shown in Figure S3, even for the non-binding case, the value of α 24 hours after medium replacement (α_{24}) is less than 2. For the non-binding case α_{24} depends only on the diffusion co-efficient (Table S3). Thus, for efficacy testing of non-ECM binding autocrine factors, it is necessary and sufficient to test the concentration range between the average concentration obtained by ELISA, and 2 (3) times this concentration for D = 10^{-10} m^2/s (D = 0.5x10^{-10} m^2/s).

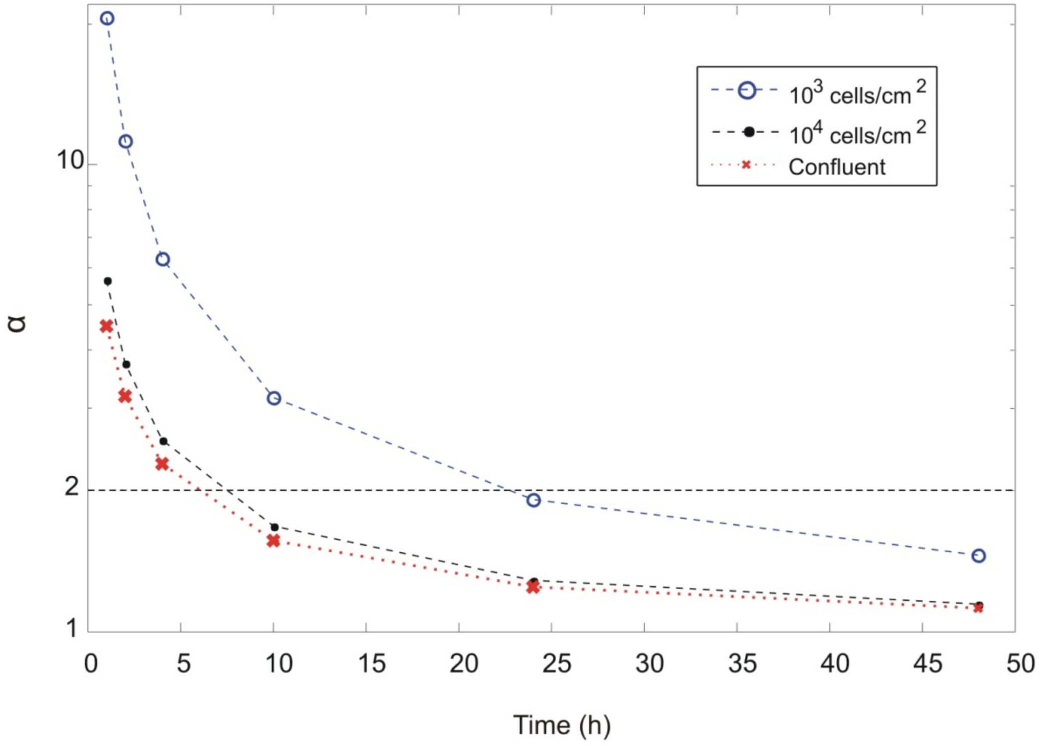

Figure S3: Value of α (Cell surface concentration/Average ligand concentration) versus time for cells plated at various densities, in the absence of ligand binding.

Table S3: Values of α_{24} for two values of the diffusion co-efficient. The diffusion co-efficient for most cytokines is in the range 0.5-1x10^{-10} m^2/s (He, L. *et al.* Biotechnol Prog 19, 544-8 (2003)).

Diffusion Co-efficient (m^2s^{-1})	Plating density (cm^{-2})	
	1000	10000
0.5x10^{-10}	2.82	1.57
1x10^{-10}	1.91	1.29

Finally, for maximally activated i.e. confluent cultures it is possible to derive analytic expressions for the concentration. I will focus on the non-binding case since, as mentioned above, it provides an upper bound on the gradient value. For a confluent culture the problem reduces to a 1-dimensional diffusion problem.

$$u_t - D\,u_{xx} = 0 \text{ for } 0 < x < h,\ t > 0$$
$$u(x,0) = 0 \qquad (1)$$
$$Du_x(0,t) = -r,\ Du_x(h,t) = 0$$

Let us convert the inhomogeneous boundary conditions to homogeneous ones.

Let $V(x,t) = -r/D(x - x^2/2h)$; then $V_x = -r/D(1- x/h)$ (2)
Then $DV_x(0) = -r,\ V_x(h) = 0$ (3)

Let $v(x,t) = u(x,t) - V(x,t)$ (4)

Then $v_t - D\,v_{xx} = (u_t - D\,u_{xx}) - (V_t - D\,V_{xx}) = 0 - (0 - r/h) = r/h$ (5)
$v(x,0) = u(x,0) - V(x,0) = 0 + r/D(x - x^2/2h) = r/D(x - x^2/2h)$ (6)
$v_x(0,t) = v_x(h,t) = 0$ (7)

Let $v(x,t) = \Sigma_{n=0}^{\infty}\, v_n(t)\, cos(n\pi x/h)$ (8)

Substituting in (5), we get
$\Sigma_{n=0}^{\infty}\ (\partial/\partial t)v_n(t)\, cos(n\pi x/h) + D(n\pi/h)^2\, \Sigma_{n=0}^{\infty}\, v_n(t)\, cos(n\pi x/h) = r/h$ (9)

Multiplying by $cos(m\pi x/h)$ and integrating from $x = 0$ to $x = $ h,
and using $\int_0^h cos(n\pi x/h)\, cos(m\pi x/h)\, dx = h/2\, \partial_{mn} + h/2\, \partial_{0n}$ yields

$\Sigma_{n=0}^{\infty}\ \{(\partial/\partial t)v_n(t) + D(n\pi/h)^2\, v_n(t)\}.(h/2\, \partial_{mn} + h/2\, \partial_{0n}) = r/h\, \partial_{m0}$ (10)

I.e. for $m = 0$ we get $(\partial/\partial t)v_0(t) = r/h$. So $v_0(t) = (r/h)\, t + c_0$. (11)

for $m > 0$, $(\partial/\partial t)v_m(t) + D(n\pi/h)^2\, v_m(t) = 0$. I.e. $v_m(t) = v_m(0)\, \exp(-D(m\pi/h)^2\, t)$. (12)

Since $v_0(0) = 0$, the initial condition (6) implies that
$v_n(0) = 2r/h \int_0^h (x - x^2/2h)\, cos(n\pi x/h)\, dx\ = -2r/Dh\, (h/\, n\pi)^2\ \ (n>0)$, (13)

and $c_0 = rh/(3D)$.

Then from (4),

$$u = v + V$$
$$= (r/h)\, t + rh/(3D) - 2rh/(D\pi^2)\,(\Sigma_{n=1}^{\infty} \exp(-D(n\pi/h)^2\, t)\, cos(n\pi x/h)/n^2) - r/D(x - x^2/2h) \quad (14)$$

Let us now try to estimate α. For the cell surface concentration, we set $x = 0$.

Then $u(0,t) = (r/h)\, t + rh/D\, (1/3 - 2/(\pi^2)\, \Sigma_{n=1}^{\infty} \exp(-D(n\pi/h)^2\, t)/n^2\,)$

For t = 24h (8.64e4 s), the value of the integral is ~ 6.7e-3 for $h = 2.5$ mm and $D = 10^{-10}$ m^2/s (evaluated numerically in Mathematica).

Next, the spatial average value of $u(x,t)$ is rt/h.

Then at $t = 24$h, $\alpha < 1.24$, and at $t = 48$h, $\alpha < 1.12$ which agree with the above results (Table S3). Similarly, for $D = 0.5\text{x}10^{-10}$ m^2/s, at $t = 24$h, $\alpha < 1.48$, and at $t = 48$h, $\alpha < 1.24$.
