# Supplementary material for: Cell Surface Concentrations and Concentration Ranges for Testing In Vitro Autocrine Loops and Small Molecules
Source: PLoS One. 2012 Dec 28;7(12):e51796. doi: 10.1371/journal.pone.0051796 (PMC3532204; doi:10.1371/journal.pone.0051796)
Supplement: Text S4 — Slowly diffusing substance added to a culture. (PDF) [file pone.0051796.s006.pdf]

Text S4: Slowly diffusing substance added to a culture

For slowly diffusing molecules, say, with a diffusion co-efficient of 5×10^{-11} m^2/s (typically large proteins with a molecular weight of around 200 kDa), the gradients can be large for the purely diffusive condition, and thus an exact calculation that includes convective transport is required. However, in the case that very little of the exogenously added substance is depleted (<10%), the gradient is indeed shallow ($0.87 < \alpha_{24} < 1$, Table S4), where α_{24} is the ratio of the cell surface concentration to the average concentration 24 hours after feeding/passaging. I.e. in this case the cell surface concentration and average concentration differ by only 13% at the most.

Table S4: Values of α_{24} for a plating density of 1000 cells/cm^2 and diffusion co-efficient = 5×10^{-11} m^2/s. The uptake rate was taken to be kc_s, where c_s is the concentration of the added substance at the surface of the cell.

k (s^{-1})	α_{24}	% substance remaining in the medium after 24 hours
1e-6	0.87	92%
1e-5	0.36	70%
1e-4	0.05	60%
